# The Late Mr. Frederick Lawrance

**Published:** 1915-09-18

**Authors:** 


### THE LATE MR. FREDERICK LAWRANCE.

Longevity is a subject of fascinating interest to many and of some interest to most people. What bearing institutional work has on the expectation of life depends upon circumstances. We can understand that for the leisured classes, for retired men enjoying their well-earned *otium cum dignitate*, participation in the management of useful institutional work may prove a comfort well calculated to prolong life. Given an equable temperament, with a tact demanded by the office, longevity should not be adversely affected by holders of administrative institutional offices. But with the executive department, from the necessities of the work, the influence must be adverse. Doctors, to use the well-understood generic term, are not a long-lived race, in contradistinction to their brethren of the cloth, the parsons. A notable exception to the rule was Mr. Frederick Lawrance, who died recently in his eighty-fifth year, and was one of the oldest practising members of his profession. The second son of the rector of Bleadon, Mr. Lawrance became M.R.C.S. in 1857 and L.S.A. in 1859, practising in Hammersmith and its neighbourhood, working almost up to the day of his death, and attending his last case of midwifery this year! A man of happy temperament, with a dignity of the old school, Mr. Lawrance enjoyed for many years an extensive practice, radiating over Kew and Putney Bridges and through Kensington Gate, which has been long since removed. Mr. Lawrance attended at Chiswick House, Chiswick, and, among others, the Duchess of Sutherland and the Marchioness of Bute were his patients. He was an example of longevity in a member of the medical profession. But he was an example—this time true to type—of generous financial carelessness. We are told he troubled little about financial affairs, never pressing for payment of accounts! And in the evening of his days, in the main owing to a co-trustee making away with a large amount in a trust estate, he found himself embarrassed, which necessitated a meeting of his friends. This meeting provided a remarkable testimonial of the esteem in which Mr. Lawrance was held, and the *res angusta domi* were not permitted to weigh down his old age. Favoured in many ways, finance was not his lucky star. As a young man Mr. Lawrance bought a half-share in a practice for £1,000, and six years later, on his partner's death, he acquired the other half, by agreeing to pay an annuity of £100 per annum to his deceased partner's three maiden sisters. The eldest sister was seventy-five. In about five years the youngest one died, the second a year later. But the eldest sister lived until she was 101 years old! Do we not all know that an annuity is a great contributing cause to longevity? The price of the second half-share was probably worth some £600 at the outside, so that Lawrance paid £2,000 too much under his kindly meant but unbusinesslike arrangement in favour of his partner's sisters. Parents who are wise provide some business training for their sons in early youth, no matter what calling in life the sons may select to follow. An annuity charged on his practice is the last thing a practitioner should agree to, for as age advances earning power decreases, fashions change, and the doctor's earnings, even in the case of eminent consultants, have a way of terminating with almost dramatic suddenness.

